# COMP report: CPQR technical quality control guidelines for treatment planning systems

**DOI:** 10.1002/acm2.12268

**Published:** 2018-01-27

**Authors:** Jose E. Villarreal‐Barajas

**Affiliations:** ^1^ Department of Physics and Astronomy University of Calgary Calgary Canada; ^2^ Present address: Medical Physics Department Royal Devon and Exeter Hospital – NHS Foundation Trust Exeter United Kingdom

**Keywords:** quality control, radiotherapy, treatment planning system

## Abstract

The Canadian Organization of Medical Physicists (COMP), in close partnership with the Canadian Partnership for Quality Radiotherapy (CPQR) has developed a series of Technical Quality Control (TQC) guidelines for radiation treatment equipment. These guidelines outline the performance objectives that equipment should meet in order to ensure an acceptable level of radiation treatment quality. The TQC guidelines have been rigorously reviewed and field tested in a variety of Canadian radiation treatment facilities. The development process enables rapid review and update to keep the guidelines current with changes in technology. This article contains detailed performance objectives and safety criteria for Treatment Planning Systems (TPS) for External Beam Radiotherapy.

## 1 INTRODUCTION

The Canadian Partnership for Quality Radiotherapy (CPQR) is an alliance among the national professional organizations involved in the delivery of radiation treatment in Canada: the Canadian Association of Radiation Oncology (CARO), the Canadian Organization of Medical Physicists (COMP), and the Canadian Association of Medical Radiation Technologists (CAMRT). Financial and strategic backing is provided by the Canadian federal government through the Canadian Partnership Against Cancer (CPAC), a national resource for advancing cancer prevention and treatment. The mandate of the CPQR is to support the universal availability of high‐quality and safe radiotherapy for all Canadians through system performance improvement and the development of consensus‐based guidelines and indicators to aid in radiation treatment program development and evaluation.

This document contains detailed performance objectives and safety criteria for *Treatment Planning Systems (TPS) for External Beam Radiotherapy*. This report focus on the basic functionally of modern TPS, it aims to provide guidelines in the form of quality control tests and their associated performance criteria.

This report is largely based on a previous document produced by J. Van Dyk published in February 2012.^1^ Please refer to the overarching document *Technical Quality Control Guidelines for Canadian Radiation Treatment Centres* 2 for a programmatic overview of technical quality control, and a description of how the performance objectives and criteria listed in this document should be interpreted. This overall process is based on prior work by Dunscombe et al.^3^

The development of the individual TQC guidelines, this one included, is spearheaded by expert reviewers and involves broad stakeholder input from the medical physics and radiation oncology community.^4^ It is the responsibility of the supervising physicist to ensure that locally available test equipment and procedures are sufficiently sensitive to establish compliance with the criteria specified within the suite of TQC guidelines.

## 2 SYSTEM DESCRIPTION

A treatment planning system (TPS) is typically comprised of: a means for inputting patient data (such as a digitizer or an interface to a CT scanner); a computer which performs the dose calculation; and finally, a means of outputting the results of the calculations, the image, and the geometrical data, which are all elements used as the basis for the calculations. The quality of a patient's treatment depends critically on both, the intrinsic accuracy of the TPS and on the correct use of the system and interpretation of the output. Acceptable quality control of a TPS inevitably includes both quality control of the system and the processes involved in its use.

The accuracy of the dose calculations themselves depends on two independent subsystems. The first is the calculation algorithm. If photon and electron transport are handled correctly under all the clinical conditions encountered then the algorithmic component can be regarded as satisfactory. However, even the most accurate algorithm will generate inaccurate dosimetry predictions if the clinical radiation beams are not accurately modeled. Recommendations do exist for tolerances on photon beam modeling.^5^, ^6^

Even if an accurate algorithm and accurate beam models are employed, the system can be misused resulting in serious detriment to patients.^7^ Such misuse often arises from a lack of understanding of the basis of the calculations, and in particular, issues to do with dose normalization. It is important to recognize that a random dosimetric or transcription error at the planning stage will be transmitted through the whole course of a patient's treatment. A systematic problem with the algorithm, beam model, or understanding of the use of the system has the potential to affect a cohort of patients.^8^ Due to the critical and central nature of the TPS in the treatment process, extensive commissioning and quality control are essential. Given the complex interdependence between the system and operators, the quality control process must extend to a detailed review on a per‐patient basis. Intensity‐modulated radiation therapy (IMRT), and most recently intensity‐modulated arc therapy (IMAT), and adaptive radiotherapy (ART), are particularly specialized and resource‐intensive applications of treatment planning and delivery systems. Quality control as it relates to IMRT, IMAT, and ART will be covered in separate Technical Quality Control (TQC) guideline documents (available at www.cpqr.ca).

The complexity of TPSs and the processes and interactions which surround them require a more detailed discussion than can be given here. The focus of this document is contained in Tables 1 and 2 and their associated notes which specify the routine quality control standards to be followed. More detailed descriptions of TPSs and in particular, commissioning activities and quality assurance, can be found in the source document^9^ and other related references.^10^, ^11^, ^12^, ^13^, ^14^, ^15^

**Table 1:** Quarterly quality control tests

Designator	Test	Performance	
		Tolerance	Action
**Quarterly**			
QTPS1	CPU/server	Functional	
QTPS2	Digitizer (if it is used clinically)	2 mm	3 mm
QTPS3	Electronic plan transfer	Data integrity	
QTPS4	Plan details	Data integrity	
QTPS5	Plotter/printer	2 mm	3 mm
QTPS6	Backup recovery	Functional	
QTPS7	CT geometry/density	2 mm/0.02	3 mm/0.03

**Table 2:** Annual quality control tests

Designator	Test	Performance	
		Tolerance	Action
**Yearly**			
ATPS1	Revalidation	Data	
ATPS2	Independent quality control review	Complete	

It should be noted that specialized TPSs are sometimes used for specific applications. Examples include high dose rate (HDR) brachytherapy, low dose rate (LDR) brachytherapy with radioactive seeds, stereotactic radiosurgery, helical tomotherapy, and Gamma Knife. In such cases where a specialized TPS is used, the principles of quality assurance and quality control espoused in this report, although modified for the specific situation, should be applied. It is worth noting that the rapid evolution of radiation treatment technologies presents significant challenges on the quality assurance and quality control of TPSs. Some of these challenges are related to the dose optimization processes and their corresponding algorithms (as used in IMRT), dose reconstruction, four‐dimensional calculations, treatments and all their associated phantoms, and quality assurance tools.^15^, ^16^

## 3 RELATED TECHNICAL QUALITY CONTROL GUIDELINES

In order to comprehensively assess the TPS performance, additional guideline tests, as outlined in related CPQR TQC guidelines must also be completed and documented, as applicable. Related TQC guidelines, available at cpqr.ca, include:

- CT Simulators
- Data Management Systems
- Safety Systems
- Major Dosimetry Equipment
- Medical Linear Accelerators and Multileaf Collimators.

Any new or upgraded TPS or its components (i.e., dose calculations algorithms, MLC modeling, etc.) should be validated against standard test cases as used for the original TPS commissioning.

## 4 TPS TEST TABLES

The following test tables are divided into quarterly and annual quality control tests. These tests are designed to cover the minimum recommendations for a clinical TPS. The tests are mainly derived from the International Atomic Energy Agency Technical reports series no. 430.^9^

### 4.A Quarterly tests notes

**Table:** 

QTPS1	For workstations: On rebooting the system, onscreen messages must be checked for indications of possible system malfunction (see more information in International Atomic Energy Agency [IAEA] Technical Reports Series No. 430 section 10.2.1^9^). The system should be checked if error message is displayed during the rebooting process. Document the error message and fix. For a multiple‐user TPS running on a server(s), rebooting may not be recommended. However, server log files shall be reviewed and error messages investigated and fixed as required. After a software or hardware upgrade of the workstations or server(s) associated with the TPS, a quarterly quality control shall be performed. Revalidation is required for a major upgrade (e.g., involving a new operating system or a new user‐server(s) communication platform)
QTPS2	Using the onscreen ruler, check that a known contour has been digitized accurately (see more information in IAEA Technical Reports Series No. 430 section 10.2.2^9^)
QTPS3	Using a standard set of at least three clinical plans covering a range of treatment configurations (photons and electrons), confirm that the data are accurately transferred from the TPS to the therapy machine (see more information in IAEA Technical Reports Series No. 430 section 10.2.11^9^)
QTPS4	Using a standard set of at least three clinical plans covering a range of treatment configurations, confirm that the data are accurately transferred from the TPS to hard copy, or digital copy for paperless environments (see more information in IAEA Technical Reports Series No. 430 section 10.2.10^9^)
QTPS5	Check the dimensions on the printout against the inputted contour and previous plots. The tolerance of 2 mm and action level of 3 mm do not apply to instances where the printout is used for patient/beam setup verification (i.e., stereotactic radiosurgery where significantly more stringent criteria are required) (see more information in IAEA Technical Reports Series No. 430 section 10.2.3^9^)
QTPS6	Check the integrity of data restored from recently backed up files (see more information in IAEA Technical Reports Series No. 430 section 10.2.4^9^). If this test requires a parallel TPS to be safely conducted and such parallel system is not available, a set of patient files from the clinical TPS shall be compared to the corresponding backup patient files
QTPS7	Check that the CT geometry and the relationship between CT number and density have not changed. Tolerances and action levels are specified in millimeters/relative electron density. Under some circumstances, for example, volumes in close proximity to the optic nerve, tighter performance criteria may be necessary (see more information in IAEA Technical Reports Series No. 430 section 10.2.6^9^)

### 4.B Annual tests notes

**Table:** 

ATPS1	Check the constancy of external beam dose calculations using a standard set of at least four clinical plans covering a range of geometries, energies, and modalities (see more information in IAEA Technical Reports Series No. 430 section 10.2.8^9^). For each type of plan, the testing shall include the most extreme scenarios likely to be encountered clinically. As part of the constancy check, the repeatability of the calculated dose‐volume histogram (DVH) shall be reviewed. Consistency between calculated percentage depth doses (PDD) and tissue‐phantom ratios (TPR), open, blocked, and wedged fields dose profiles shall be compared with the corresponding beam data used for the TPS commissioning. Calculation must be performed using the clinical mode of the TPS. Test the treatment planning process from end‐to‐end under the most realistic circumstances: CT scan and plan an anthropomorphic phantom using the immobilization devices used clinically. Treat the phantom in clinical mode including usual imaging; verify the measured to plan‐dose agreement by comparing it with baseline TPS commissioning data
ATPS2	To ensure redundancy and adequate monitoring, a second qualified medical physicist must independently verify the implementation, analysis, and interpretation of the quality control tests at least annually

## CONFLICT OF INTEREST

The author declares no conflict of interest.
